# Development and Molecular Characterization of 55 Novel Polymorphic cDNA-SSR Markers in Faba Bean (*Vicia faba* L.) Using 454 Pyrosequencing

**DOI:** 10.3390/molecules18021844

**Published:** 2013-01-31

**Authors:** Sundan Suresh, Jong-Hyun Park, Gyu-Taek Cho, Ho-Sun Lee, Hyung-Jin Baek, Sok-Young Lee, Jong-Wook Chung

**Affiliations:** National Agrobiodiversity Center, National Academy of Agricultural Science, Rural Development Administration, Suwon 441-853, Korea

**Keywords:** cDNA-SSR, genetic diversity, 454 sequencing, *Vicia faba*

## Abstract

Faba bean (*Vicia faba* L.) is a major food source and fodder legume, popularly known for its high content of seed-protein. Its role is critical in crop rotation, and for fixing nitrogen effectively. Polymorphic simple sequence repeat markers from transcript sequences (cDNA; simple sequence repeat [SSR]) were developed for faba bean (*Vicia faba*). We found that 1,729 SSR loci from 81,333 individual sequence reads and 240 primer pairs were designed and synthesized. In total, 55 primer pairs were found to be polymorphic and scorable consistently when screened in 32 accessions. The number of alleles ranged from 2 to 15, frequency of major alleles per locus varied from 0.17 to 0.91, the genotypes number ranged from 2 to 17, observed and expected heterozycosity values ranged from 0.00 to 0.44 and 0.17 to 0.89 and overall PIC values ranged from 0.16 to 0.88 respectively. These markers will be a useful tool for assessing the genetic diversity, understanding the population structure, and breeding patterns of faba bean.

## 1. Introduction

Faba bean (*Vicia faba* L.) is currently the third most important winter season food legume globally. Faba bean represent an important source for dietary protein to human beings, edible oil and animal feed. Its critical role in crop rotation, effective nitrogen fixation, soil improvement abilities, and contribution to reducing energy input costs have long been recognized. Faba bean is a diploid with 2n = 2x = 12 chromosomes, is partially cross pollinated (ranging from 4 to 84%) and possesses one of the largest genomes among crop legumes (~13,000 Mb) [1,2]. Despite being an alternative source of protein for human and effective in nitrogen fixation, the number of molecular markers available for faba bean is still scarce, with only 100 microsatellite (simple sequence repeat; SSR) markers [3,4] and only 32 EST–SSR markers [5,6] having been reported. The development of more reliable and informative molecular markers needs to be improved to enhance our understanding about the faba bean.

Next-generation transcriptome sequencing is an efficient means to generate superior resources for the development of cDNA–simple sequence repeat (SSR) markers. cDNA-SSR markers present some intrinsic advantages over genomic SSRs in their direct association with transcribed genes, low expense for development cost, and higher level of transferability to related species [7] and cDNA-SSRs, are highly polymorphic, rather than EST-derived SSR markers [8]. In a recent study, the authors sequenced faba bean transcriptomes using 454 pyrosequencing technologies and found that 1,729 SSR loci from 81,333 individual sequence reads and limited number of sequence 240 were submitted to GenBank, which paved the way for microsatellite marker development. In our study, we developed and characterized polymorphic cDNA-SSR markers based on these sequences for *V. faba* to facilitate the studies on molecular diversity of this species.

## 2. Results and Discussion

The *V. faba* transcriptome sequencing yielded 29.61 Mb and GS De Novo yielded 81,333 raw sequencing reads, based on the GS-FLX sequencer. SSR is one of the most popular marker systems, consisting of varying numbers of tandem repeated di-, tri-, or tetra-nucleotide DNA motifs. To identify SSR markers, we used the ARGOS program with default settings for the *V. faba* unigene collections. In total, 1,729 potential SSR motifs were identified, and the majority belonged to trinucleotide (67.61%) and dinucleotide (19.08%) repeats. All other types of SSRs such as tetra-, penta-, and hexa- nucleotide motifs were relatively low (13.3%), and the majority of trinucleotide SSRs had the GAA/AAG/AGA motif, followed by those with the TGG/CGT/GGT motif, and others with the CTT/TTC/TCT motif. The GA/AG, AT/TA, and GT/TG motifs were identified among the dinucleotide cDNA-SSRs. The relative proportion of SSR motif types in faba bean [9] were observed as similar to that of other plant species [10,11,12]. 

Among the identified SSR loci, we selected 240 sequences that were deposited in GenBank (GenBank accession number: KC218573–KC218812). Of the 240 primer pairs, only 55 primer pairs produced consistently amplified (Table 1). These 55 cDNA-SSR loci were screened in 32 accessions. The number of alleles (*N_A_*) per locus varied widely among the markers (Table 2), ranged from 2 to 15, with an average of 6.0 alleles. The frequency of major alleles (*M_AF_*) per locus varied from 0.17 to 0.91 with an average of 0.563, the genotypes number (*N_G_*) ranged from 2 to 17, with an average of 6.3. The *H_O_* values were ranged from 0.00 to 0.44 with an average of 0.074, the *H_E_* values were ranged from 0.17 to 0.89 with an average of 0.587 and overall *PIC* values ranged from 0.16 to 0.88, with an average of 0.550 respectively. Similar observation was also found in the *Vicia faba* [6]. These cDNA-SSR markers were developed in our study are found to be a useful tools for further studies on molecular diversity and population structure of faba bean.

**Table 1:** Characteristics of the 55 cDNA-SSR markers for faba bean (*Vicia faba* L.).

Locus	Primer sequences (5′–3′)	Product Size	Modif	GenBank Accession No.		Ta (°C)		BLAST top hit accession no.			Description	*E-value*
GBSSR-VF-8	F:TAAAGCAGCTCCGGATGAR:TCGGTGGAGGAGTTGTTG	242	(TTG)5	KC218580		58		None			None	None
GBSSR-VF-19	F:TCCATCAACCTCAAATCCA R:CCGTACTTGTCCACGGAA	201	(CAA)5	KC218591		58		XP_003637109.1			hypothetical protein MTR_072s1002 [*Medicago truncatula*]	1.00E-41
GBSSR-VF-20	F:TCCACCAAGTCCACCTGAR:AATAAGGGCGCAGGAGAG	198	(GTG)6	KC218592		58		None			None	None
GBSSR-VF-21	F:CGAAGCCTCCTCCTCTTCR:TGGTGTTGTATTCGGGGA	199	(CCT)4	KC218593		58		ABF66654.1			EBP1 [*Ammopiptanthus mongolicus*]	3.00E-37
GBSSR-VF-22	F:CGAAGCCTCCTCCTCTTCR:CAAGTGGCCGTTTTTCAA	271	(CCT)4	KC218594		58		None			None	None
GBSSR-VF-32	F:CGAAGCCTCCTCCTCTTCR:GGTGTTGATTCGGGGAGT	197	(CCT)4	KC218604		58		XP_003614315.1			hypothetical protein MTR_5g048060 [*Medicago truncatula*]	5.00E-44
GBSSR-VF-34	F:CGGGAACCAACTCAACAAR:CCACCACCGCAACTATGT	186	(CGA)4	KC218606		58		None			None	None
GBSSR-VF-38	F:ACCATTTGGCCTGTTCCTR: CGCTACCCAAATGCTGAA	226	(GTG)6	KC218610		58		CAA61492.1			phloem specific protein [*Vicia faba*]	7.00E-18
GBSSR-VF-52	F:GGTTTCTTGTCCAAATAAGACGR:TGCGATTCTGGAAATTGG	261	(CAG)5	KC218624		58		AFW76468.1			putative protein phosphatase 2A family protein [*Zea mays*]	5.00E-27
GBSSR-VF-84	F:CGGCGTCTAGAACGTTTGR:AACTAGCGCAGCTCATCG	243	(GCG)5	KC218656		58		XP_003555733.1			PREDICTED: ammonium transporter 1 member 1-like isoform 1 [*Glycine max*]	1.00E-40
GBSSR-VF-113	F:TGGTGGTGCTTCTTTCCAR:TGGTGAGCTTGGAACTGC	213	(CTT)5	KC218685		59		XP_002524296.1			Abscisic stress ripening protein, putative [*Ricinus communis*]	4.00E-05
GBSSR-VF-115	F:TGCTGCTTTTCCAACCATR:GTGCATGCCATAACAAAA	177	(AT)7	KC218687		57		XP_003600578.1			NAD(P)H-quinone oxidoreductase subunit N [Medicago truncatula]	0.018
GBSSR-VF-119	F:GTGGCCTGTACTGGTGGAR: ACTCGTTGGGGCTAGGAA	225	(GAA)5	KC218691		58		AFK37381.1			unknown [*Medicago truncatula*]	1.00E-80
GBSSR-VF-131	F:CCGTACTAAATGAAGCCTTTR:GGCAATCAAGTCCGGTAA	238	(TA)6	KC218703		57		XP_003546500.1			RNA polymerase-associated protein CTR9 homolog [*Glycine max*]	1.00E-05
GBSSR-VF-149	F:ACGACATGGTGATGAATCCTR:ACGTGACCGAGTGACGAC	211	(CAA)8	KC218721		58		XP_003610732.1			hypothetical protein MTR_5g006400 [*Medicago truncatula*]	1.00E-58
GBSSR-VF-153	F:TCCCGACGCTACTTCTCAR:CCGAGATCTGCAAACAGC	225	(CCA)5	KC218725		58		AFK44330.1			unknown [*Medicago truncatula*]	3.00E-44
GBSSR-VF-154	F:ACACCAATGTTTTTGCGGR:TCCTGACTTTGCTGAGGC	247	(GAA)5	KC218726		58		XP_003522646.1			uncharacterized protein LOC100777431 [*Glycine max*]	1.00E-22
GBSSR-VF-159	F:GTGCCATCATCCTCGAAAR:CAGCTGCTAGGTTGCCTG	235	(TCT)4	KC218731		58		ABD32307.2			Nuclear factor related to kappa-B-binding protein, related [*Medicago truncatula*]	3.00E-61
GBSSR-VF-164	F:ACCATTTGGCCTGTTCCTR:CAAGGAGGGTTGTTTACGA	199	(GTG)6	KC218736		57		CAA61492.1			phloem specific protein [*Vicia faba*]	3.00E-11
GBSSR-VF-168	F:TCTCCAAACCCTCCTCGTR:TCAGCCACAAAATCAGCA	250	(TGT)6	KC218740		57		XP_003605197.1			GRAS family transcription factor [*Medicago truncatula*]	9.00E-05
GBSSR-VF-172	F:CGGTTTCTAAATCTGGCGR:GCTCCATTGAAACCAATTCT	232	(TTG)5	KC218744		57		XP_003552039.1			uncharacterized protein LOC100790537 [*Glycine max*]	1.00E-15
GBSSR-VF-173	F:CACAGACAGGTTTCGGGAR:TTGGGTGCAACATCATCA	247	(CAC)6	KC218745		58		XP_003607781.1			Low-temperature inducible [*Medicago truncatula*]	7.00E-10
GBSSR-VF-175	F:TGCCATTCCATCTGAACCR:CCAGGCAATGGAATCTGA	256	(TCC)7	KC218747		58		ABN08705.1			DDT; Homeodomain-related [*Medicago truncatula*]	1.00E-16
GBSSR-VF-180	F:TTGGGTGCAACATCATCAR:CGGGAAAGAATCAGAGGC	230	(GTG)4	KC218752		58		XP_003607781.1			Low-temperature inducible [*Medicago truncatula*]	0.002
GBSSR-VF-184	F:CCATCAACGGAGGACTCAR:TAGGGGAAACAGGGGCTA	183	(TGC)4	KC218756		58		XP_003594716.1			Prefoldin subunit [Medicago truncatula]	2.00E-56
GBSSR-VF-185	F:GTTTCTTGTCCAAATAAGACGR:CCTGACACTACACGAAAGAC	184	(CAG)5	KC218757		58		ACJ83506.1			unknown [*Medicago truncatula*]	0.47
GBSSR-VF-190	F:TTTTATGAAAGCGAAACCGR:TGGACAAAGGCAACAATCA	156	(TCT)5	KC218762		58		XP_003608427.1			Myocyte-specific enhancer factor 2B [*Medicago truncatula*]	3.00E-17
GBSSR-VF-203	F:TTCCCTGACCTTTCAGCAR:TGTTGGGCACCTCAAGTT	231	(CTC)4	KC218775		58		XP_003555847.1			CAX-interacting protein 4-like [*Glycine max*]	2.00E-14
GBSSR-VF-209	F:CCATCAACGGAGGACTCAR:GGGAACAGGGGCTAGAGA	181	(TGC)4	KC218781		58		XP_002519249.1			prefoldin subunit, putative [*Ricinus communis*]	3.00E-28
GBSSR-VF-213	F:CCAGGTTTCTTCCTCCGAR:TTTAATTTTGGGCCGGAT	271	(TC)7	KC218785		58		None			None	None
GBSSR-VF-215	F:ACAAACTGAGTCCAACCATGTR:TGCCACTGCTTCTCTTGG	279	(CAA)4	KC218787		58		XP_003638360.1			Cytochrome b reductase [*Medicago truncatula*]	1.00E-79
GBSSR-VF-216	F:CCCATTCAGAACGTGGAAR:GGCTGCAATCTACCACCA	194	(ACA)4	KC218788		58		ACM50914.1			aquaporin [*Medicago falcata*]	9.00E-18
GBSSR-VF-218	F:GCTCCGCATATACAAAGATGR:ATGGCGGTGGTCACTATG	211	(CCA)5	KC218790		57		None			None	None
GBSSR-VF-220	F:ACCATTTGGCCTGTTCCTR:CAAATGCTGAAATGGCCT	217	(GTG)6	KC218791		58				CAA61492.1	phloem specific protein [*Vicia faba*]	2.00E-13
GBSSR-VF-221	F:CCGAAATGAAGATGATGATGAR:TGAAAGGGAAACTGAAAGTCA	243	(CAT)5	KC218792		58				None	None	None
GBSSR-VF-237	F:TTGGGTGCAACATCATCAR:CGGGAAAGAATCAGAGGC	234	(GTG)5	KC218793		58				AKF45200.1	unknown [*Medicago truncatula*]	2.00E-05
GBSSR-VF-245	F:TTGTACCAAGCATTTAATTTTCR:CATTACCACCGTGAGGCA	279	(TCA)5	KC218794		57				None	None	None
GBSSR-VF-255	F:GGAGATGCTTTTGGCCTCR:TGGTCCTGCAGTTTCCAT	262	(GTT)5	KC218795		58				XP_003544671.1	uncharacterized protein LOC100809022 [*Glycine max*]	2.00E-24
GBSSR-VF-258	F:AAACCCTCCATCTTCGGAR:CAGGAGAGTATCTTGATAAGGC	165	(GTG)4	KC218796		58				CAB16318.1	cysteine proteinase precursor [*Vicia narbonensi*s]	5.00E-30
GBSSR-VF-262	F:TCTGGCGAGTGGCATACTR:GCCTTCTACACAACGGCTT	224	(GAA)5	KC218797		58				CAB07811.1	sucrose transport protein [Vicia faba]	9.00E-14
GBSSR-VF-263	F:ATGCCACCCTCACTTTCCR: TCCTTCCAAATTCAGAATCC	157	(ACA)6	KC218798		58				XP_003592083.1	Polypyrimidine tract-binding protein-like protein [*Medicago truncatula*]	2.7
GBSSR-VF-266	F:CAAATGCAATGCTGCAAAR:AACAGGTGGTGGCTGATG	292	(TCA)5	KC218799		58				None	None	None
GBSSR-VF-270	F:CAGGGATTGCACACAACAR:TGAAAGGAATGGAAGAGGG	252	(TCA)4	KC218800		58				AAD50628.1	alpha-tubulin [*Gossypium hirsutum*]	0.017
GBSSR-VF-271	F:TTTTATGAAAGCGAAACCGR:TTGGAACAAAGGCAACAA	158	(TCT)5		KC218801		56		ACJ84526.1		unknown [*Medicago truncatula*]	1.00E-05
GBSSR-VF-276	F:GCTCTTCAACCTGCCCTTR:GGGACAGTTGCTGTTGGA	150	(CAA)4		KC218802		58		AAU11490.1		monodehydroascorbate reductase I [*Pisum sativum*]	9.00E-55
GBSSR-VF-285	F:AAGAAGGTGTCGCGGAAGR:CCGCACCTTCTCCTCTCT	176	(GAT)5		KC218803		58		None		None	None
GBSSR-VF-288	F:GCCTGTGGCTAGAAGCAAR:TAATGGTCCCAGCACCTC	237	(TCA)5		KC218804		57		None		None	None
GBSSR-VF-293	F:CCCATTCAGAACGTGGAAR:TCAGCAATAAAAGCTCTTGGA	160	(ACA)4		KC218805		58		ACU17538.1		unknown [*Glycine max*]	0.018
GBSSR-VF-295	F:CAGGGTTACGATTGCTCGR:AAATGGCAAGAGATTAAAAGCA	175	(TGC)4		KC218806		58		XP_003594716.1		Prefoldin subunit [*Medicago truncatula*]	3.00E-35
GBSSR-VF-297	F:AGACCAAGAATCAAGGTCACAR:CTCTTCACAAAGCGACCCT	150	(AAT)6		KC218807		58		CAB39664.1		putative protein [*Arabidopsis thaliana*]	4.00E-05
GBSSR-VF-298	F:AATCATCCGGAACCATCCR:GACGTCTGAGGAGAGGGC	249	(TTC)4		KC218808		58		XP_003624491.1		hypothetical protein MTR_7g083930 [*Medicago truncatula*]	3.00E-25
GBSSR-VF-311	F:GGCCTTTCAACAAGAGGGR:ACCATTTGGCCTGTTCCT	207	(CAC)6		KC218809		58		CAA61492.1		phloem specific protein [*Vicia faba*]	2.00E-16
GBSSR-VF-312	F:GGCTATGGTGGTCATGGAR:TTACGCCGCCTCCAC	151	(TGG)5		KC218810		58		CAH40798.1		putative glycine rich protein precursor [*Pisum sativum*]	6.00E-08
GBSSR-VF-319	F:CATGCATTTGCTGCTCAAR:GTGCAGGCACTACATGGG	158	(TTG)5		KC218811		58		NP_001237269.1		VHS and GAT domain protein [*Glycine max*]	5.00E-28
GBSSR-VF-323	F:TCTGCTTCCATCTTCATCGR:TTGCTGAAGTTGCTCTGTGA	180	(ACA)4		KC218812		58		None		None	None

*Note*: T_a_, annealing temperature.

**Table 2:** Diversity statistics from initial primer screening in 32 accessions of faba bean (*Vicia faba* L.)*.*

Marker	*M_AF_*	*N_G_*	*N_A_*	*H_O_*	*H_E_*	*PIC*
GBSSR-VF-8	0.17	17	15	0.28	0.89	0.88
GBSSR-VF-19	0.50	6	6	0.00	0.66	0.61
GBSSR-VF-20	0.42	8	9	0.09	0.74	0.71
GBSSR-VF-21	0.38	6	6	0.06	0.74	0.70
GBSSR-VF-22	0.47	7	8	0.03	0.71	0.67
GBSSR-VF-32	0.66	5	5	0.00	0.54	0.51
GBSSR-VF-34	0.65	6	6	0.00	0.56	0.53
GBSSR-VF-38	0.69	7	7	0.10	0.49	0.46
GBSSR-VF-52	0.47	7	7	0.00	0.69	0.65
GBSSR-VF-84	0.58	4	4	0.00	0.60	0.56
GBSSR-VF-113	0.38	7	7	0.00	0.76	0.73
GBSSR-VF-115	0.34	10	9	0.03	0.79	0.76
GBSSR-VF-119	0.28	6	6	0.00	0.80	0.77
GBSSR-VF-131	0.30	8	8	0.06	0.80	0.78
GBSSR-VF-149	0.47	9	9	0.00	0.73	0.70
GBSSR-VF-153	0.50	4	4	0.00	0.67	0.62
GBSSR-VF-154	0.47	7	7	0.06	0.71	0.68
GBSSR-VF-159	0.41	5	5	0.00	0.70	0.65
GBSSR-VF-164	0.52	8	7	0.06	0.67	0.64
GBSSR-VF-168	0.25	10	11	0.13	0.81	0.78
GBSSR-VF-172	0.30	18	14	0.44	0.85	0.84
GBSSR-VF-173	0.52	7	7	0.09	0.66	0.63
GBSSR-VF-175	0.23	13	12	0.25	0.82	0.80
GBSSR-VF-180	0.50	5	5	0.09	0.66	0.61
GBSSR-VF-184	0.61	5	5	0.03	0.56	0.51
GBSSR-VF-185	0.91	3	3	0.06	0.17	0.17
GBSSR-VF-190	0.56	9	9	0.28	0.64	0.61
GBSSR-VF-203	0.91	2	2	0.00	0.17	0.16
GBSSR-VF-209	0.77	4	4	0.03	0.39	0.35
GBSSR-VF-213	0.47	5	5	0.00	0.69	0.64
GBSSR-VF-215	0.72	3	3	0.00	0.44	0.39
GBSSR-VF-216	0.75	2	2	0.00	0.38	0.30
GBSSR-VF-218	0.81	2	2	0.00	0.30	0.26
GBSSR-VF-220	0.68	4	4	0.06	0.50	0.46
GBSSR-VF-221	0.45	7	6	0.13	0.68	0.63
GBSSR-VF-237	0.63	6	5	0.09	0.56	0.53
GBSSR-VF-245	0.72	5	5	0.00	0.45	0.42
GBSSR-VF-255	0.75	4	4	0.00	0.41	0.37
GBSSR-VF-258	0.56	6	6	0.13	0.63	0.60
GBSSR-VF-262	0.44	11	10	0.25	0.74	0.71
GBSSR-VF-263	0.41	8	8	0.19	0.70	0.65
GBSSR-VF-266	0.59	4	4	0.00	0.58	0.53
GBSSR-VF-270	0.69	3	3	0.00	0.48	0.43
GBSSR-VF-271	0.38	10	8	0.22	0.74	0.70
GBSSR-VF-276	0.72	3	3	0.00	0.44	0.40
GBSSR-VF-285	0.73	5	5	0.26	0.45	0.43
GBSSR-VF-288	0.64	5	5	0.03	0.53	0.49
GBSSR-VF-293	0.78	3	3	0.00	0.35	0.31
GBSSR-VF-295	0.70	5	5	0.03	0.47	0.43
GBSSR-VF-297	0.59	8	6	0.16	0.59	0.55
GBSSR-VF-298	0.72	3	3	0.00	0.44	0.40
GBSSR-VF-311	0.72	7	6	0.09	0.45	0.42
GBSSR-VF-312	0.69	2	2	0.00	0.43	0.34
GBSSR-VF-319	0.80	4	4	0.03	0.35	0.32
GBSSR-VF-323	0.66	7	6	0.23	0.52	0.49
Mean	0.563	6.3	6.0	0.074	0.587	0.550

(M_AF _), Major allele frequency; (N_G_), number of genotype; (N_A_), number of allele; (H_O_), observed heterozygosity; (H_E_), expected heterozygosity; (PIC), polymorphic information content.

## 3. Experimental

### 3.1. Plant Material

Faba bean seeds were selected from the National Agrobiodiversity Center, Rural Development Administration, Suwon, Korea. Seedlings were germinated and grown in a glasshouse. The leaves of young seedlings were used to extract the mRNA required to synthesize the cDNA library and for 454 sequencing.

### 3.2. cDNA Preparation

Total RNA was extracted from *V. faba* leaves that were frozen in liquid nitrogen, ground well into a powder, and then extracted using an RNeasy Plant Mini kit (Qiagen, Valencia, CA, USA) following the manufacturer’s instructions. The integrity of total RNA was determined using a BIOSPEC-NANO spectrophotometer (Shimadzu, Kyoto, Japan) and agarose gel electrophoresis. mRNA was purified using the PolyATract mRNA Isolation System IV (Promega, Madison, WI, USA), and the purified products were used to synthesize full-length cDNAs using a ZAP-cDNA Synthesis kit (Stratagene, Santa Clara, CA, USA). Finally, cDNA was fragmented by nebulization for library construction.

### 3.3. Library Preparation

Approximately 1 µg of cDNA was used to generate a DNA library to use with the rapid library preparation method manual (Roche Life Science Inc., Branford, CT, USA). The cDNA fragment ends were polished (blunted), and two short adapters were ligated to both ends according to standard procedures described previously. The adapters provided priming of the sequences for both amplification and sequencing of the sample library fragments, as well as the sequencing key, a short sequence of four nucleotides used by the system’s software for base calling. Following repair of any nicks in the double-stranded library, the unbound strand of each fragment was released (with 5-Adaptor A). Finally, the quality of this single-stranded template DNA library was assessed using a 2100 BioAnalyzer (Agilent, Waldbronn, Germany). The library was quantified to determine the optimal amount of the library needed as input for emulsion-based clonal amplification.

### 3.4. 454 Pyrosequencing

Single effective copies of template species from the DNA library to be sequenced were hybridized to DNA capture beads. The immobilized library was then resuspended in an amplification solution, and the mixture was emulsified, followed by polymerase chain reaction (PCR) amplification. The DNA carrying beads were recovered from the emulsion and enriched after amplification. The second strands of the amplified products were melted, leaving the amplified single-stranded DNA library bound to the beads. The sequencing primer was then annealed to the immobilized amplified DNA templates. After amplification, a single DNA carrying bead was placed into each well of a PicoTiterPlate (PTP) device. Simultaneous sequencing with multiple samples on a single PTP (four region gasket) was used. The PTP was then inserted into the FLX Genome Titanium sequencer for pyrosequencing [13,14], and sequencing reagent was sequentially flowed over the plate. Information from the PTP wells was captured simultaneously by a camera, and the images were processed in real-time by an onboard computer. Multiplex identifiers were used to specifically tag unique samples in a GS FLX Titanium sequencing run, which were recognized by the GS data analysis software after the sequencing run and provided high confidence for assigning individual sequencing reads to the correct sample. Sequence assembly was performed after sequencing using GS De Novo Assembler software (Roche) to produce contigs and singletons. All sequence data were conformed to references using GS Reference Mapper software (Roche). 

### 3.5.Discovery of cDNA-SSR Markers

All contigs and singletons from both transcriptomes were then used to mine SSR motifs, and the SSR motifs were identified using the ARGOS pipeline program (version 1.46) at the default settings to survey the molecular markers present in the *V. faba* accessions [15]**.** Parameters were designed for identifying perfect di-, tri-, tetra-, penta-, and hexa-nucleotide motifs with a minimum of six repeats. Primer design parameters were set as follows: length range, 18–23 nucleotides with 21 as optimum; PCR product size range, 100–400 bp; optimum annealing temperature, 55 °C; and GC content 40–60%, with 50% as optimum. Faba bean genomic DNA was extracted from 18 diverse faba bean accession samples for EST-SSR marker validation using a DNeasy® Plant Mini kit (Qiagen, Valencia, CA, USA), according to the manufacturer’s instructions. Fresh leaf tissue from each accession was used for each extraction and ground well using liquid nitrogen. DNA was resuspended in 100 μL water, and dilutions were made to 10 ng/μL followed by storage at either −20°C or −80°C. Randomly selected EST-SSR primer pairs were validated experimentally, and forward primers were synthesized by adding the M13 sequence to enable fluorescent tail addition through the PCR amplification process [16]. PCR conditions included a hot-start at 95 °C for 10 min, followed by 10 cycles at 94 °C for 30 s, 60–50 °C for 30 s and 72 °C for 30 s, followed by 25 cycles at 94 °C for 30 s, 50 °C for 30 s, and 72 °C for 30 s, and a final elongation step of 72 °C for 10 min. PCR products were separated and visualized using the QIAxcel Gel Electrophoresis System (Qiagen).

### 3.6. Data Analysis

These 55 SSR loci were screened in 32 accessions (Table 3). The number of alleles (*N_A_*), major allele frequency (*M_AF_*), observed heterozygosity (*H_O_*), Expected heterozygosity (*H_E_*), number of genotype (*N_G_*), and polymorphism information content (*PIC*) were calculated using GenAlEx (version 6.5) [17]. 

**Table 3:** List of faba bean (*Vicia faba* L.) accessions and details of collection sites.

No.	Temp. ID	ARS No.	Origin
1	K193517	PI 221516	Afghanistan
2	K193518	PI 223418	Iran
3	K193519	PI 234634	Australia
4	K193520	PI 251331	Israel
5	K193521	PI 253806	Iraq
6	K193522	PI 254920	Spain
7	K193523	PI 275641	Ethiopia
8	K193524	PI 284338	Lebanon
9	K193525	PI 284345	Italy
10	K193526	PI 319901	Soviet Union
11	K193527	PI 358270	Serbia and Montenegro
12	K193528	PI 371806	United Kingdom
13	K193529	PI 415027	France
14	K193530	PI 415050	Sudan
15	K193531	PI 430133	Egypt
16	K193532	PI 442559	BEL
17	K193533	PI 458504	Mexico
18	K193534	PI 469135	Japan
19	K193535	PI 469144	Cyprus
20	K193536	PI 469180	Greece
21	K193537	PI 469182	Jordan
22	K193538	PI 469189	India
23	K193539	PI 478506	Bolivia
24	K193540	PI 499959	China
25	K193541	PI 577723	Kyrgyzstan
26	K193542	PI 577735	Chile
27	K193543	PI 577741	Nepal
28	K193544	PI 655323	Morocco
29	K193545	PI 655325	Peru
30	K193546	PI 655326	Ecuador
31	K193547	PI 655330	Kenya
32	K193548	PI 655344	Pakistan

Temp ID, Korean Gene Bank ID; ARS No, USDA-ARS Number.

## 4. Conclusions

In our study we have developed 55 cDNA-SSR markers, and they were successfully used to investigate the genetic diversity among 32 accessions of faba bean. However, there seems to be a relatively higher genetic diversity within *V. faba*, as only 55 of 240 cDNA-SSR loci exhibited polymorphism. The availability of co-dominant polymorphic cDNA-SSR markers provided a tool set for further study on molecular diversity, and will greatly facilitated the genetic structure studies of *V. faba* populations, the identification and conservation of faba bean.
